# Genome evolution and long-term demographic history in true crocodiles

**DOI:** 10.1093/g3journal/jkag178

**Published:** 2026-08-05

**Authors:** Ana M Saldarriaga-Gómez, Agusto Luzuriaga-Neira, Mario Vargas-Ramírez, George Amato, Evon Hekkala

**Affiliations:** Department of Biological Sciences, Fordham University, Bronx, NY 10458, United States; American Museum of Natural History, NewYork, NY 10024, United States; Grupo de Biodiversidad y Conservación Genética, Instituto de Genética, Universidad Nacional de Colombia, Bogot, Cundinamarca 111321, Colombia; Department of Ornithology, American Museum of Natural History, NewYork, NY 10024, United States; Grupo de Biodiversidad y Conservación Genética, Instituto de Genética, Universidad Nacional de Colombia, Bogot, Cundinamarca 111321, Colombia; American Museum of Natural History, NewYork, NY 10024, United States; Department of Biological Sciences, Fordham University, Bronx, NY 10458, United States; American Museum of Natural History, NewYork, NY 10024, United States

**Keywords:** *Crocodylus*, genome assembly, demographic history, dN/dS, comparative genomics, molecular evolution

## Abstract

Reference-quality genomes remain scarce for true crocodiles (*Crocodylus*), limiting comparative analyses of genome evolution and demographic history. Here, we generated and analyzed 2 long-read genomes, 1 for *Crocodylus intermedius* and 1 for *C. niloticus*, to investigate genome architecture, coalescent effective population size (*N*_e_), and patterns of molecular evolution across crocodilians. Comparative analyses revealed broadly similar repeat landscapes in both species and extensive macro-synteny with *Alligator sinensis*, indicating strong structural conservation across crocodilian genomes. Using phased diploid assemblies and MSMC2, we reconstructed historical *N*_e_ trajectories and found marked differences between species. *Crocodylus intermedius* exhibited persistently low *N*_e_ throughout most of the late Quaternary, with a pronounced decline during the Late Pleistocene–early Holocene transition. In contrast, *C. niloticus* showed substantially larger *N*_e_ over comparable time intervals. Genome-wide codon-based analyses identified significant heterogeneity in *d*_N_/*d*_S_ (ω) among crocodilian lineages. *Crocodylus niloticus* showed the lowest genome-wide ω, whereas elevated values in *C. intermedius* and other lineages were consistent with reduced long-term efficacy of purifying selection under smaller historical population sizes. Branch-site tests identified candidate genes under positive selection in both focal species, with functional categories related to ion transport, endocrine regulation, and cellular signaling. Together, these results provide genomic resources for *Crocodylus* and support an association between long-term demographic history and genome-wide patterns of molecular evolution across crocodilians.

## Introduction

Crocodiles (family Crocodylidae) are large, semi-aquatic predators distributed throughout the tropical regions of Asia, Australia, Africa, and the Americas. Within the family, 3 extant genera are recognized (*Crocodylus*, *Osteolaemus*, and *Mecistops*), with *Crocodylus* being the most diverse genus, currently comprising 13 recognized species (Meredith et al. 2024). Despite their ecological importance as top predators and wide geographic distribution, genomic resources remain unevenly developed across the family, substantially limiting comparative and evolutionary analyses. To date, no published reference-quality long-read genome is available for *Crocodylus*. Existing published resources rely primarily on short-read assemblies, including those generated for *Crocodylus porosus* (Schneider, 1801) by Ghosh et al. (2020) and *Crocodylus rhombifer* (Cuvier, 1807) by Meredith et al. (2024). However, short-read assemblies are often highly fragmented and lack reliable long-range structural information. Their use as mapping references can introduce alignment ambiguities, particularly in repetitive or duplicated regions, biasing estimates of coverage, heterozygosity, and demographic history (Treangen and Salzberg 2011; Li and Durbin 2011). These limitations have hindered the development of a robust genomic framework for comparative studies across *Crocodylus* and key evolutionary questions within the genus remain unresolved. For example, phylogenetic and phylogenomic studies of Neotropical *Crocodylus* species have yielded conflicting results regarding species relationships and divergence patterns (e.g. Milián-García et al. 2018a, 2018b, 2020). More broadly, the lack of reference-quality genomes has restricted the ability to investigate how genome structure, demographic history, and molecular evolution have shaped diversification within the group. Whole-genome sequencing using long-read technologies provides the most powerful framework for addressing these questions.

Comparative studies suggest that crocodilian genomes have evolved slowly over the past million years relative to other tetrapods. This pattern is supported by consistently low genome-wide ratios of nonsynonymous substitutions (*d*_N_) to synonymous substitutions (*d*_S_), which indicate pervasive long-term purifying selection and reduced rates of protein evolution (Yang and Bielawski 2000; Green et al. 2014; Figuet et al. 2016). This signal has been proposed as a genomic correlate of intrinsic extinction risk, as lineages with low substitution rates and limited adaptive turnover may exhibit reduced evolutionary responses to rapid environmental change (Luzuriaga-Neira and Alvarez-Ponce 2022). Furthermore, because the efficacy of purifying selection depends strongly on effective population size (*N*_e_), genome-wide *d*_N_/*d*_S_ ratios can serve as an indirect proxy for long-term *N*_e_ (Figuet et al. 2016; Luzuriaga-Neira and Alvarez-Ponce 2022). Persistently low *d*_N_/*d*_S_ values reflect strong selective constraint and historically large *N*_e_, whereas elevated ratios may indicate reduced efficiency of selection associated with demographic decline (Ohta 1992; Charlesworth 2009). Understanding how these slow evolutionary dynamics interact with historical changes in *N*_e_ therefore provides a valuable framework for investigating how long-term genomic stability is maintained while still permitting adaptation to changing environmental conditions in crocodilians.

In this study, we generate and analyze 2 reference-quality genomes of true crocodiles: 1 for *Crocodylus intermedius* (Graves, 1819) and 1 for *Crocodylus niloticus* (Laurenti, 1768), providing a foundational genomic framework for the genus. Specifically, we aim to (1) assemble and annotate the genomes, (2) reconstruct the demographic history of both species, and (3) investigate patterns of molecular evolution and genome architecture.

## Materials and methods

### Genome assembly and annotation

#### Sampling and lab work

The 2 focal species analyzed in this study, *Crocodylus intermedius* and *C. niloticus*, represent ecologically and evolutionarily distinct lineages within *Crocodylus*. *C. intermedius* is a critically endangered Neotropical crocodile endemic to the Orinoco Basin of Colombia and Venezuela, where it inhabits large river systems and associated floodplain environments (Medem 1981; Seijas et al. 2010). The species experienced severe population declines during the twentieth century due to overexploitation and habitat degradation and is currently considered one of the most threatened crocodilians worldwide (Medem 1981, Balaguera-Reina et al. 2018). In contrast, *C. niloticus* is a broadly distributed African crocodile currently classified as Least Concern by the IUCN, occupying freshwater ecosystems across much of sub-Saharan Africa and exhibiting a substantially wider geographic distribution and ecological breadth than *C. intermedius* (Shirley et al. 2014; IUCN Crocodile Specialist Group 2023b). Phylogenetically, *C. niloticus* represents the sister lineage to the American crocodile clade, within which *C. intermedius* occupies the most basal position among extant species, consistent with the transoceanic dispersal scenarios proposed for the evolutionary diversification of *Crocodylus* (Oaks 2011; Milián-García et al. 2020).

Sequencing was conducted using 2 blood samples, 1 from *Crocodylus intermedius* and 1 from *C. niloticus*. The *C. intermedius* sample came from a wild-born individual from the Cravo Norte River in Colombia and is currently part of the captive-breeding program at the Estación de Biología Tropical Roberto Franco (EBTRF) in the Meta Department, Colombia. This sample is deposited in the DNA and Tissue Bank (BTBC) of the Instituto de Genética, Universidad Nacional de Colombia under the accession number UNAL:BTBC:11172. The *C. niloticus* sample was collected in 2001 from the Tana River in Kenya as part of an IUCN crocodile specialist group monitored harvest program (sample Kenya Tana3; Hekkala et al. 2010, 2011). High-molecular-weight genomic DNA was extracted for both samples using the QIAGEN MagAttract HMW DNA Kit following the manufacturer's protocols. DNA quantity and integrity were assessed using the Agilent TapeStation system. Genome sequencing was performed using the PacBio Revio platform, generating HiFi reads with 1 SMRT Cell per sample. Laboratory work for *C. intermedius* sample was carried out at the Instituto de Genética, Universidad Nacional de Colombia (Bogotá, Colombia), and *C. niloticus* sample was processed at the Institute of Comparative Genomics, American Museum of Natural History (New York, USA).

#### Genome assembly

Raw PacBio HiFi reads quality was assessed using a *k*-mer–based approach. *k*-mer frequency distributions were generated using Jellyfish v2.3.0 (Marçais and Kingsford 2011) to estimate average sequencing coverage, genome size, and genome-wide heterozygosity in Genomescope2.0 (Ranallo-Benavidez et al. 2020). *De novo* genome assemblies were generated using Hifiasm v0.25.0 (Cheng et al. 2021). Hifiasm performs phased assembly by leveraging heterozygous variants to separate homologous chromosomes, producing 3 assemblies per sample: a primary assembly, which represents a collapsed, high-contiguity reference sequence suitable for downstream analyses; and 2 haplotype-resolved assemblies (haplotype 1 and haplotype 2), which correspond to the alternative parental haplotypes and capture allelic variation across the genome. This strategy allows the generation of a high-quality genome assembly while explicitly representing heterozygosity when present.

Genome completeness was assessed using BUSCO (Benchmarking Universal Single-Copy Orthologs) v5.5.0 (Simão et al. 2015), which evaluates the presence of highly conserved single-copy orthologous genes expected to be present in a complete genome. BUSCO analyses were conducted using vertebrata_odb10 and sauropsida_odb10 datasets. Second, standard assembly contiguity and structural metrics were calculated using QUAST v5.3.0 (Gurevich et al. 2013). Finally, reference-free assembly accuracy was evaluated using Merqury v1.3 (Rhie et al. 2020), which leverages *k*-mer spectra to assess assembly quality without reliance on a reference genome. *k*-mers were counted from the HiFi reads using Meryl v1.3 (https://github.com/marbl/meryl) and compared against *k*-mers present in the assembly to estimate consensus quality value (QV), assembly completeness, and potential duplicated or collapsed regions.

Mitochondrial genomes were assembled and annotated using MitoHiFi v3.2.3 (Allio et al. 2020, Uliano-Silva et al. 2023), a pipeline optimized for the assembly of mitochondrial genomes from long-read sequencing data. Published reference mitogenomes available in GenBank (accession number NC_015648.1 for *C. intermedius* and NC_008142.1 for *C. niloticus*) were used as guides to identify mitochondrial reads within the raw PacBio HiFi datasets. The resulting mitochondrial assemblies were visualized and manually inspected in Geneious Prime v.2019.2.3 (Biomatters Ltd.). After identifying all contigs corresponding to mitochondrial DNA, these sequences were removed from the primary nuclear genome assemblies. The resulting mitochondria-filtered nuclear assemblies were subsequently used for downstream genomic analyses.

#### Genome annotation

Assemblies were soft masked using EarlGrey v6.2.0 (Baril et al. 2024) to identify and mask repetitive elements. Soft masking preserves the underlying nucleotide sequence while preventing repeats from being interpreted as protein-coding genes during annotation. Since species-specific transcriptomic data were not available for the target genomes, gene annotation was conducted using available crocodilian transcript evidence from *C. porosus* (GCA_001723895.1; Ghosh et al. 2020) and *Alligator sinensis* (Fauvel, 1879) (GCA_000455745.1; Pan et al. 2025). Homology-based gene prediction was performed using GeMoMa v1.7.1 (Keilwagen et al. 2019), which uses conserved exon–intron structures across related species to infer gene models in the absence of species-specific RNA-seq data. Duplicated gene models were filtered, and only the longest isoform per gene was retained using AGAT tools (https://github.com/NBISweden/AGAT), as annotation informed by multiple reference species was expected to generate redundancy in gene models. Gene symbols were assigned according to the nomenclature of *Gallus gallus* genome (GCA_016699485.1).

Annotation quality was assessed using BUSCO v5.5.0 sauropsida_odb10 reference datasets (Simão et al. 2015) at the proteome level, based on protein sequences translated from the coding sequences (CDS) of the final annotations. This evaluation was performed for both newly annotated genomes and for the published proteomes of *A. sinensis* and *C. porosus* as benchmarks for completeness and duplication. OMAmer v2.1.0 (Rossier et al. 2021) and OMArk v0.3.0 (Nevers et al. 2024) were used to assign predicted proteins to hierarchical orthologous groups (HOGs) to evaluate annotation completeness, taxonomic consistency, and potential contamination by quantifying the proportion of genes that are conserved, lineage-specific, missing, or assigned to distant taxa. In contrast to BUSCO, which relies on a fixed set of universal single-copy orthologs to estimate completeness, HOGs rely on a broader phylogenomic framework and provide finer-scale insight into annotation consistency, lineage representation, and possible overprediction or loss of gene models.

#### Genome architecture and synteny analyses

To evaluate genome synteny and candidate structural rearrangements, we compared the 2 assembled *Crocodylus* genomes with the chromosome-scale genome of *A. sinensis* (GCA_000455745.1; Pan et al. 2025). For our *C. niloticus* genome, since Hi-C data is not available, we performed a reference-guided scaffolding step using RagTag v2.1.0 (Alonge et al. 2022), using as reference a PacBio Hi-C genome assembly for *C. niloticus* deposited in GenBank (GCA_054825085.1). Although this reference genome is not yet associated with a publication, it provides chromosome-level scaffolding. The sample used for this reference assembly originated from a captive crocodile housed in a crocodile farm in Israel, but the original parents were from South Africa. The availability of chromosome-level assemblies for *A. sinensis* and *C. niloticus* provides information on chromosome orientation and structure, whereas the *C. intermedius* assembly remains at scaffold level.

Pairwise whole-genome alignments were generated using Minimap2 v2.30 (Li, 2018), producing PAF alignment files for each pair of genomes comprising all possible combinations. To reduce noise caused by too short or fragmented alignments for *C. intermedius*, alignment blocks were filtered such that only scaffolds larger than 5 Mb were retained, and for each *A. sinensis* and *C. niloticus* chromosome, the top 1,000 longest syntenic alignment blocks were selected. To focus on large-scale chromosomal organization, collinear alignments were merged, and only large syntenic segments were retained (>50 kb for *Alligator–Crocodylus* comparisons and >200 kb for the *C. niloticus–C. intermedius* comparison), to exclude smaller local alignments. Filtered alignments were then visualized and analyzed using the syntenyPlotteR v. 1.0.0 package in R (Quigley et al 2023), with scaffolds ordered by decreasing length within each species. We characterized candidate large-scale chromosomal rearrangements by identifying 3 types of events: (i) regions showing changes in orientation indicative of inversions; (ii) cases in which genomic regions derived from distinct *A. sinensis* chromosomes co-localized within a single *C. niloticus* chromosome; and (iii) instances where a single *A. sinensis* chromosome corresponded to multiple *C. niloticus* chromosomes. Analyses were restricted to chromosome-scale scaffolds corresponding to the 16 pseudochromosomes to avoid artifacts from unanchored scaffolds.

### Demographic history inference

We used the Multiple Sequentially Markovian Coalescent framework (MSMC2; Schiffels and Durbin 2014; Schiffels et al. 2020) to infer demographic history based on coalescence rates in both species. The method reconstructs the history of change in effective population size over time using the distribution of the most recent common ancestor between 2 alleles within an individual. Because crocodilians lack differentiated sex chromosomes, all genomic regions were treated uniformly.

For *C. niloticus*, to increase genomic representation for this species, we incorporated data from the GenBank genome (GCA_054825085.1) used previously. We used the raw reads and reassembled them following the same pipeline used for our genomes (see ***Genome assembly*** section). Using this approach, phased haplotypes were obtained directly from long-read genome assemblies generated with Hifiasm for 1 individual of *C. intermedius* and 2 individuals of *C. niloticus.* Because these assemblies explicitly separate haplotype 1 and haplotype 2, heterozygous variation is already encoded in the diploid assembly structure. To capture heterozygous differences, haplotype 2 assemblies were aligned against their corresponding haplotype 1 assemblies using Minimap2 v2.30 (Li, 2018), producing alignments that reflect local heterozygosity patterns across the genome. Scaffolds were ordered by length, and Single-Nucleotide Polymorphism (SNP) variants were identified on each scaffold using BCFtools (Danecek et al. 2021). These variants were normalized relative to the haplotype 1 reference scaffold, and variant data were converted into MSMC format using msmc-tools (https://github.com/stschiff/msmc-tools; Schiffels and Durbin 2014). Demographic inference was performed using MSMC2 (Schiffels and Durbin 2014; Schiffels et al. 2020) combining multihetsep files from all scaffolds to generate genome-wide estimates of coalescent effective population size through time. Additionally, we conducted 20 independent analyses in which 30% of the genome was randomly sampled in each replicate to assess whether the observed patterns remained consistent across randomly selected genomic subsets.

MSMC2 outputs demographic estimates in coalescent units, which were converted into interpretable values of time and effective population size. *N*_e_ was calculated from the inverse coalescence rate (λ) using standard MSMC scaling relationships. Time intervals were scaled using a generation time of 20 yrs and a mutation rate of 7.89 × 10^−9^ mutations per site per generation, based on estimates reported previously for crocodilians (Green et al. 2014; Pan et al. 2025). Confidence intervals for both time and *N*_e_ were estimated using the results obtained from the 20 independent runs.

### Molecular evolution and selection analyses

To estimate branch-specific *d*_N_/*d*_S_ ratios and detect candidate signatures of positive selection, we compiled genomic datasets for 6 crocodilian species, including our annotated genomes of *C. intermedius* and *C. niloticus*. A long read (∼17× coverage) genome of *Crocodylus siamensis* (Schneider, 1801), deposited in GenBank (GCA_052110815.1) and currently lacking an associated publication, was annotated using the previously presented pipeline and incorporated into the dataset. We also included the published assemblies of *C. porosus* (Ghosh et al. 2020) and *C. rhombifer* (Meredith et al. 2024), which were generated from short-read data. These were first scaffolded against our *C. intermedius* genome using RagTag v2.1.0 (Alonge et al. 2022) and subsequently annotated. In addition, we incorporated the published long-read genome of *A. sinensis* from Pan et al. (2025), for which we generated a curated gene annotation by removing duplicated isoforms using AGAT tools v1.6.1 (https://github.com/NBISweden/AGAT) and retaining the longest isoform per gene. The quality and redundancy of gene models in the original and curated annotations were assessed using BUSCO v5.5.0 sauropsida_odb10 reference datasets (Simão et al. 2015) at the proteome level.

Single-copy orthologous genes were identified using OrthoFinder v3.1.3 (Emms et al. 2019) based on both CDS and amino acid sequences (AA) derived from the genome annotations. Orthologous CDS and AA were aligned using MAFFT v7.525 (Katoh et al. 2002), generating 1 alignment per gene/protein. AA alignments containing more than 1 stop codon were discarded, and CDS alignments whose lengths were not multiples of 3 were removed to avoid frameshift artifacts. Codon alignments were then reconstructed using PAL2NAL (Suyama et al. 2006), which combines protein alignments with their corresponding nucleotide sequences while preserving the reading frame and removing inconsistent regions. The resulting alignments were then concatenated into a supermatrix using AMAS (Borowiec 2016).

### Genome-wide d_N_/d_S_ analyses

Rates of protein evolution were estimated using the CODEML module implemented in PAML v4.9 g package (Yang 2007). We used the previously reported phylogeny for crocodilians defined as (*A. sinensis*, (((*C. rhombifer*, *C. intermedius*), *C. niloticus*), (*C. siamensis*, *C. porosus*))) (Oaks 2011; Shirley et al. 2014; Srikulnath et al. 2015). To assess variation in evolutionary rates across the phylogeny, 2 analyses were implemented on a concatenated CDS alignment. First, the Model 0 (M0) was used to estimate a single nonsynonymous to synonymous substitution rate ratio (*ω* = *d*_N_/*d*_S_) across all branches in the tree, assuming a constant rate of protein evolution among lineages. Second, the free-ratios model (FR) was applied, allowing each branch in the phylogeny to have an independent *ω* value, thereby testing for lineage-specific variation in evolutionary rates. Prior to concatenation, outlier genes were identified and removed based on analyses conducted at the level of individual alignments, excluding those with ω values greater than 0.5 under the M0 model. Model fit was evaluated using likelihood ratio tests (LRTs) comparing the log-likelihood values (lnL) obtained for each model. Statistical significance of differences between models was assessed using a χ^2^ distribution (*P* = 0.05), with degrees of freedom equal to the difference in the number of parameters (np) between each pair of nested models. A significant result indicates heterogeneity in *d*_N_/*d*_S_ ratios among branches of the phylogeny. Separately, gene-wise branch-specific ω estimates obtained under the FR were used to compare the distribution of *d*_N_/*d*_S_ ratios among species. To minimize bias associated with unreliable synonymous substitution rate estimates, genes with *dS* values lower than 0.001 were excluded from these comparisons. Pairwise differences among species were then evaluated using paired Wilcoxon rank-sum tests, with *P*-values adjusted for multiple testing using the Benjamini–Hochberg correction.

#### Branch-site tests for positive selection

To identify candidate genes evolving under positive selection, we applied branch-site models in the CODEML module implemented in PAML v4.9 g package (Yang 2007), to the individual CDS alignments described above. In each analysis, the branch corresponding to either *C. intermedius* or *C. niloticus* was specified as the foreground lineage, while all remaining branches were treated as background. For each orthologous gene alignment Model A (which allows a class of sites to evolve under positive selection along the foreground branch) was compared with the corresponding null model (Model A null). LRTs were performed for each gene, and statistical significance was evaluated using a χ^2^ distribution (*P* = 0.01), with degrees of freedom equal to the difference in the number of parameters (np) between each pair of nested models. Genes showing significant differences between models were considered candidates for positive selection on the focal lineage.

Candidate genes were functionally annotated using their corresponding *G. gallus* ortholog gene symbols as reference identifiers (GCA_016699485.1). These were queried against the PANTHER classification system (Mi et al. 2019) to retrieve PANTHER Gene IDs, protein family and subfamily assignments, protein class annotations, and associated Gene Ontology (GO) biological process and molecular function categories (The Gene Ontology Consortium 2021). This framework was used to summarize the functional composition of candidate gene sets and to identify enriched molecular functions and biological pathways within each focal lineage.

## Results

We generated and analyzed long-read genomes for *Crocodylus intermedius* and *C. niloticus* to investigate genome structure, demographic history, and patterns of molecular evolution across crocodilians. Comparative analyses revealed highly complete and structurally conserved genomes, but substantial differences in long-term effective population size trajectories and lineage-specific evolutionary rates between species. Genome-wide analyses further identified heterogeneity in *d*_N_*/d*_S_ across crocodilian lineages and multiple candidate genes evolving under positive selection.

### Genome assembly and annotation

Using PacBio HiFi sequencing, de novo assembly, and annotation based on homology-guided gene prediction, we generated high-quality reference genomes for both species. The *k*-mer–based analysis performed for *Crocodylus intermedius* yielded an estimated haploid genome size of approximately 2.06 Gb (Supplementary Fig. 1). The genome exhibited low heterozygosity, estimated between 0.14–0.15%, indicating limited nucleotide-level variation within the sampled individual. Sequencing depth for the dataset was 48.87X coverage. For the *C. niloticus* sample, the *k*-mer–based analysis did not converge, likely due to the insufficient effective coverage of 32.63X associated with partial fragmentation of the input DNA used for sequencing. Nevertheless, this coverage level still represents high sequencing depth and is sufficient for a reference-grade genome assembly.

**Fig. 1.**
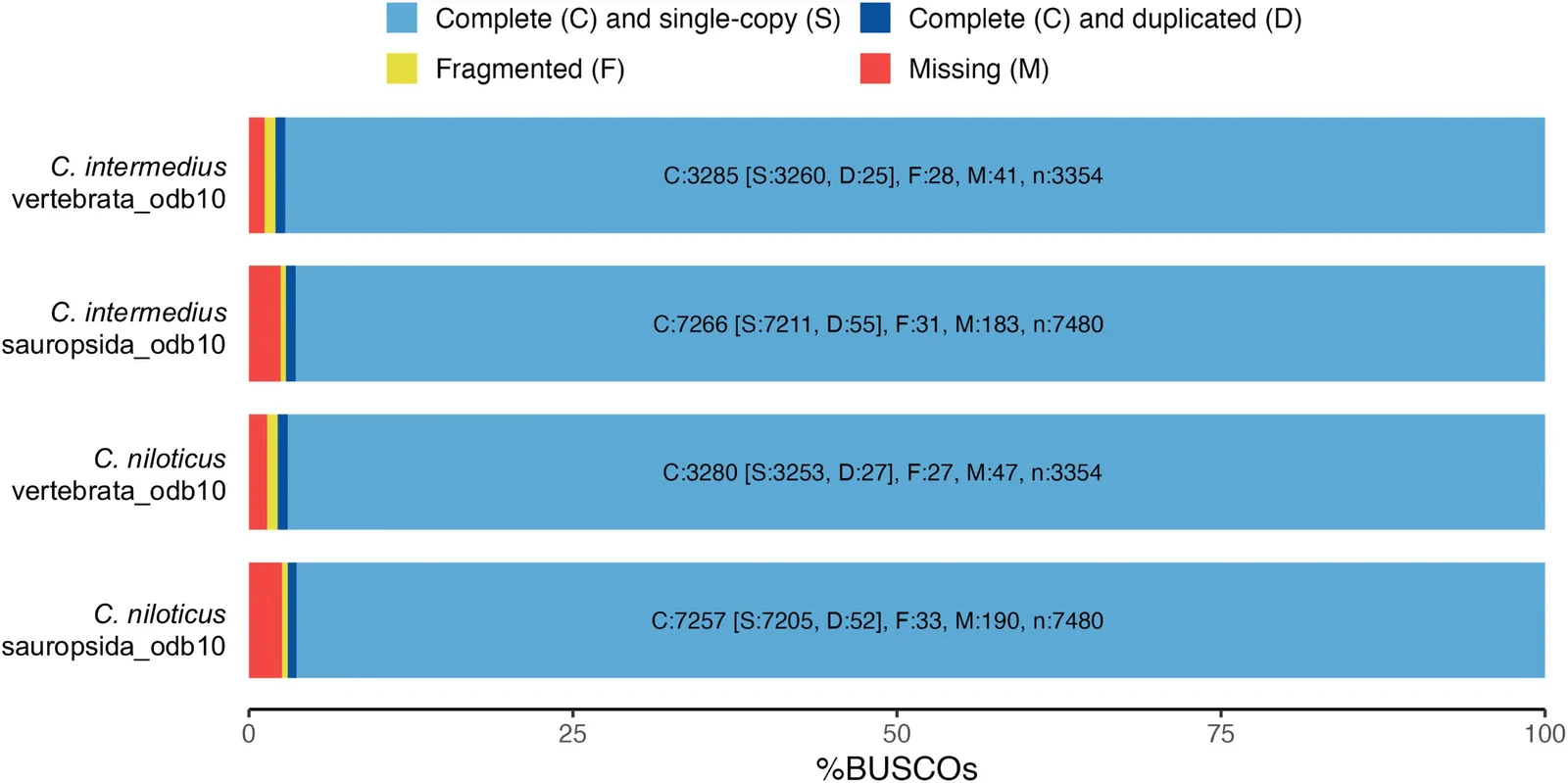
BUSCO completeness assessment of the primary genome assemblies of *Crocodylus intermedius* and *C. niloticus* using vertebrata_odb10 and sauropsida_odb10 databases.

Assembly completeness was evaluated with BUSCO on the primary assembly using sauropsida_adb10 and vertebrata_odb10 databases. Both assemblies showed high completeness, with BUSCO scores ranging from 97.0 to 97.9% in *C. intermedius* and from 97.0 to 97.8% in *C. niloticus* (Fig. 1). QUAST analysis revealed highly contiguous genomes for both species, with total assembly sizes of ∼2.35 Gb, GC content of ∼45%, N50 values of 48.7 Mb for *C. intermedius* and 61.7 Mb for *C. niloticus* (Supplementary Table 1). Meryl/Merqury analysis reported a QV of 69.24 for *C. intermedius* and an estimated error rate of 1.19 × 10^−7^, with an overall *k*-mer completeness of 99.6%. Similarly, the *C. niloticus* primary assembly showed QV values exceeding 70 and comparably low error rates. The *k*-mer spectrum further supported high accuracy and completeness of both assemblies, showing that the vast majority of *k*-mers were shared between reads and assemblies, with only minor fractions of haplotype-specific or read-only *k*-mers (Supplementary Fig. 2). Finally, the assembled mitochondrial genome length was 16,791 base pairs (bp) for *C. intermedius* and 16,425 bp for *C. niloticus*. Both mitogenomes included 13 protein-coding genes, 22 tRNAs, 2 rRNAs, as well as 1 control region (D-loop), for a total of 38 annotated elements.

Both *C. intermedius* and *C. niloticus* showed broadly similar repetitive landscape compositions (Supplementary Fig. 3). Long Interspersed Nuclear Elements (LINE) and unclassified repeats constituted the largest fractions of repetitive content, accounting for ∼38–39% of the genome. Minor differences were observed between species. *Crocodylus intermedius* showed slightly higher proportions of LINEs (18.85%) and unclassified elements (19.73%) than *C. niloticus* (17.52 and 18.56%, respectively), whereas *C. niloticus* exhibited slightly higher proportions of Long Terminal Repeats (1.84% vs 1.71%) and other low-complexity repeats (2.08% vs 0.95%). All other repeat classes occurred at low and similar frequencies in both species, and non-repetitive sequences comprised over half of each genome (54.05% in *C. intermedius* and 55.20% in *C. niloticus*).

**Fig. 2.**
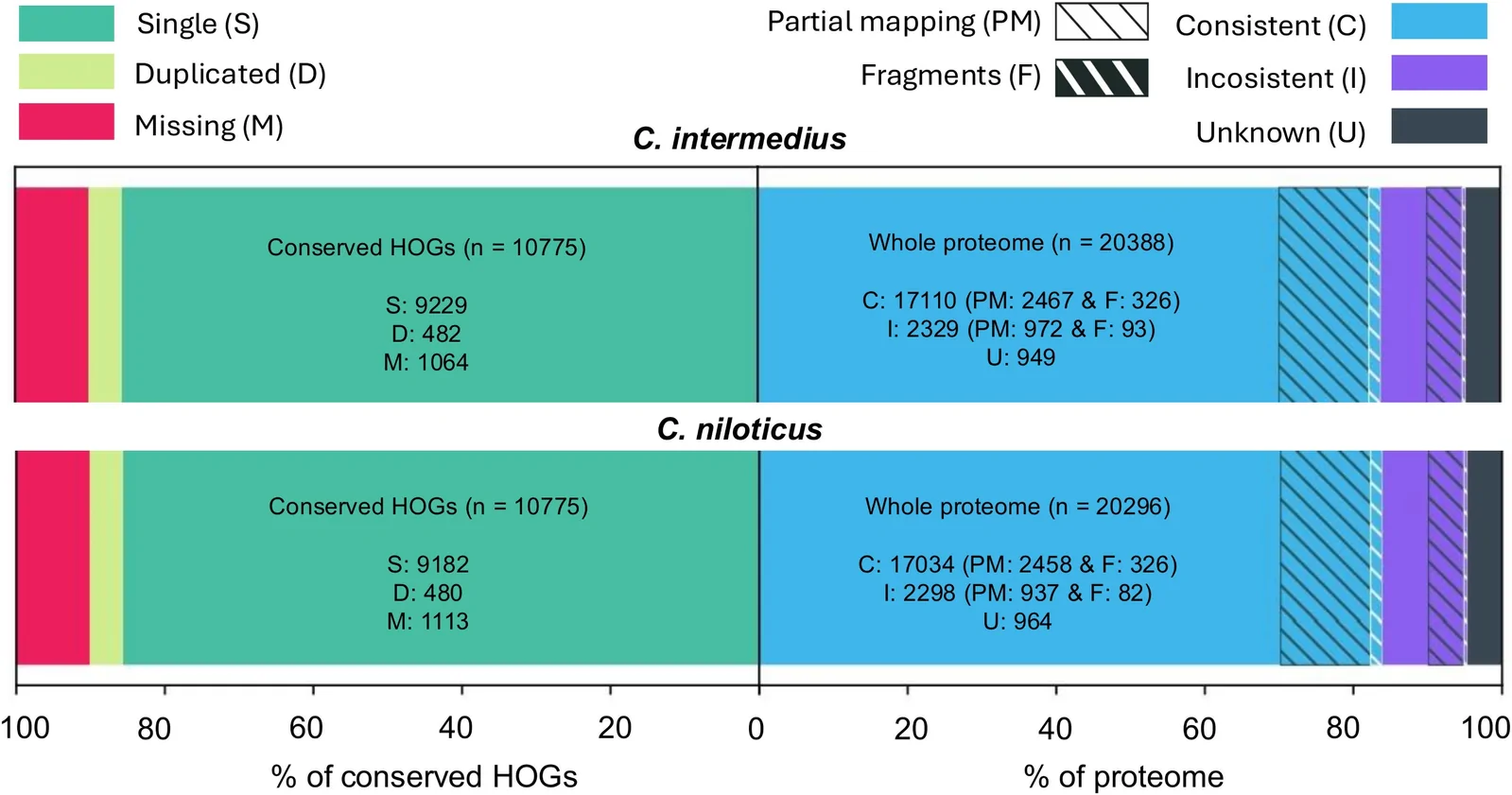
OMArk summary of orthologous group completeness and proteome consistency for *Crocodylus intermedius* and *C. niloticus* using the Archosauria clade. HOG: hierarchical orthologous groups.

Genome annotation completeness was evaluated using BUSCO analyses with the sauropsida_odb10 database and complementary OMAmer/OMArk analyses within the Archosauria clade (Supplementary Table 2; Fig. 2). The *C. intermedius* annotation recovered 88.2% complete BUSCOs, with the vast majority corresponding to single-copy genes (87.2%) and a low proportion of duplicated BUSCOs (1.0%). The *C. niloticus* annotation showed 87.8% complete BUSCOs, including 86.8% single-copy and 1.0% duplicated BUSCOs. Fragmented and missing BUSCOs accounted for less than ∼2% and ∼10% of the total, respectively. OMAmer/OMArk analyses, conducted within the Archosauria clade, further supported these results by evaluating orthologous group representation and annotation consistency across a broad phylogenomic framework (Fig. 2). For *C. intermedius*, 85.65% of conserved HOGs were classified as single copy, with 4.47% duplicated and 9.87% missing. Comparable patterns were observed for *C. niloticus*, with 85.22% single copy, 4.45% duplicated, and 10.33% missing HOGs. In both species, most duplicated HOGs corresponded to unexpected duplications (∼4.2%). BUSCO analyses of published proteomes revealed high overall completeness (>96%) but also high duplication level. The *C. porosus* proteome (Ghosh et al. 2020) showed 39.1% duplicated BUSCOs, while the *A. sinensis* proteome (Pan et al. 2025) exhibited 44.2% duplicated BUSCOs.

**Fig. 3.**
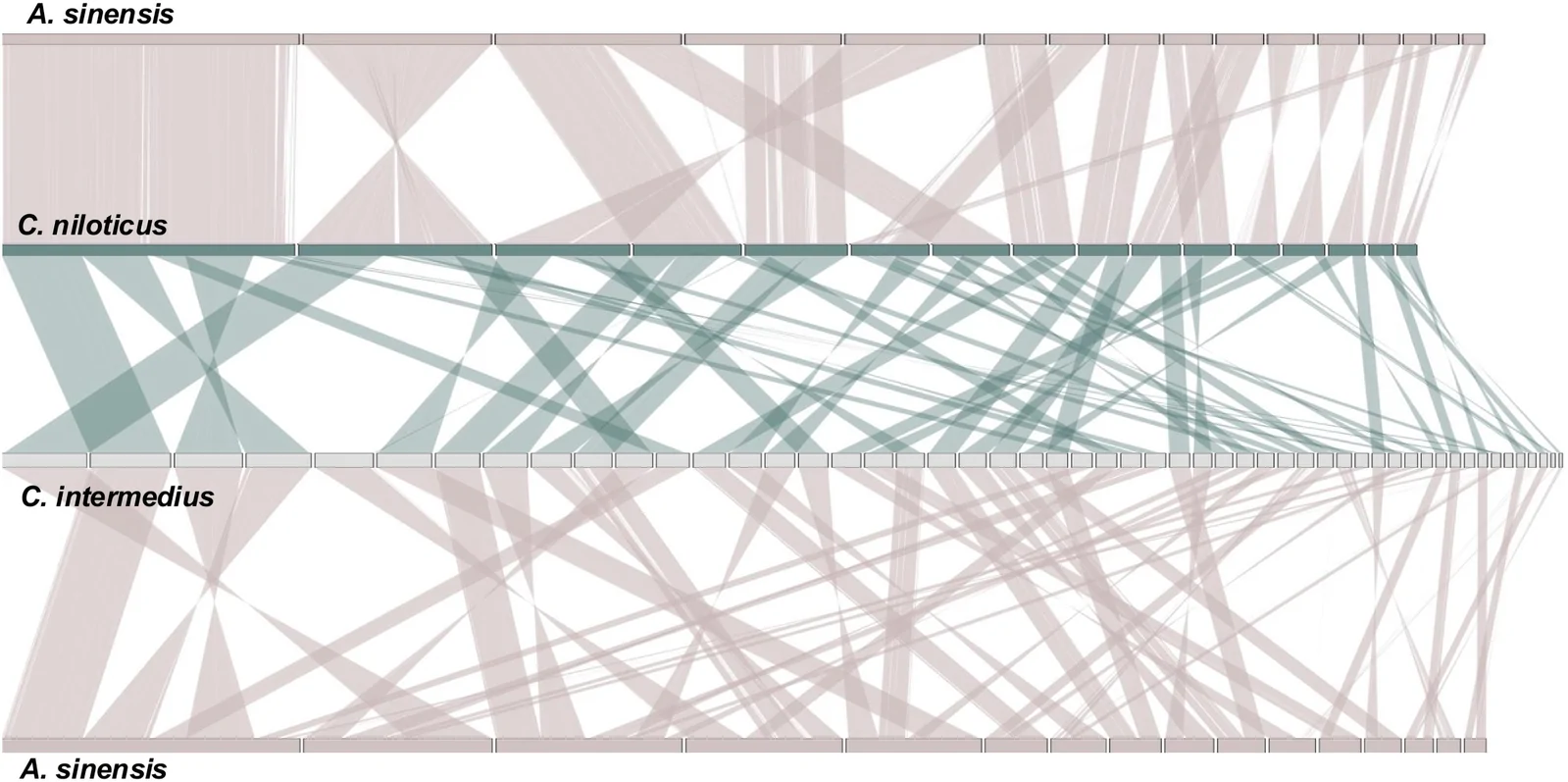
Genome-wide synteny among *Alligator sinensis*, *Crocodylus intermedius*, and *C. niloticus*. Rectangular blocks represent the 16 chromosomes of *A. sinensis* and *C. niloticus* and the 53 largest scaffolds of *C.* intermedius.

Pairwise genome comparisons of *A. sinensis* produced substantially more raw alignment hits (*A. sinensis–C. intermedius*: 172,794; *A. sinensis–C. niloticus*: 170,782) than the *C. niloticus–C. intermedius* comparison (10,675), consistent with the greater divergence from the *Alligator* genome. After filtering, we retained 312 syntenic blocks for *A. sinensis–C. intermedius*, 318 for *A. sinensis–C. niloticus*, and 280 for *C. niloticus–C. intermedius*, with median block sizes ranging from 3.38 to 4.24 Mb. We identified multiple signatures of candidate chromosomal rearrangements between *A. sinensis* and *C. niloticus*, including both interchromosomal breakpoint events and orientation changes (Fig. 3; Supplementary Tables 5 and 6). Chromosomal fusions were detected for 4 *A. sinensis* chromosomes (chromosomes 1, 7, 11, and 15, with 1, 2, 3, and 2 events, respectively), whereas fissions were detected for chromosomes 3 and 11 (1 and 2 events, respectively). In addition, numerous inversion breakpoints were identified across different chromosomes in both genomes, particularly affecting *C. niloticus* chromosomes 1 and 6, and *A. sinensis* chromosome 7n (Fig. 3; Supplementary Table 5). Detailed genomic coordinates of candidate breakpoint regions and their associated rearrangement types, including inversion and breakpoint events with or without orientation change are provided in Supplementary Table 6.

### Demographic history inference

Using phased haplotypes derived from long-read assemblies and MSMC2 coalescent analyses (Schiffels and Durbin 2014), we reconstructed historical effective population size trajectories for both species using a generation time of 20 yrs and a mutation rate of 7.89 × 10^−9^ mutations per site per generation, based on estimates reported previously for crocodilians (Green et al. 2014; Pan et al. 2025). We conducted SNP calling within each individual yielding 290,305 SNPs for *C. intermedius*, 3,140,053 SNPs for our *C. niloticus* genome and 3,621,354 SNPs for the GCA_054825085.1 *C. niloticus* genome.

**Fig. 4.**
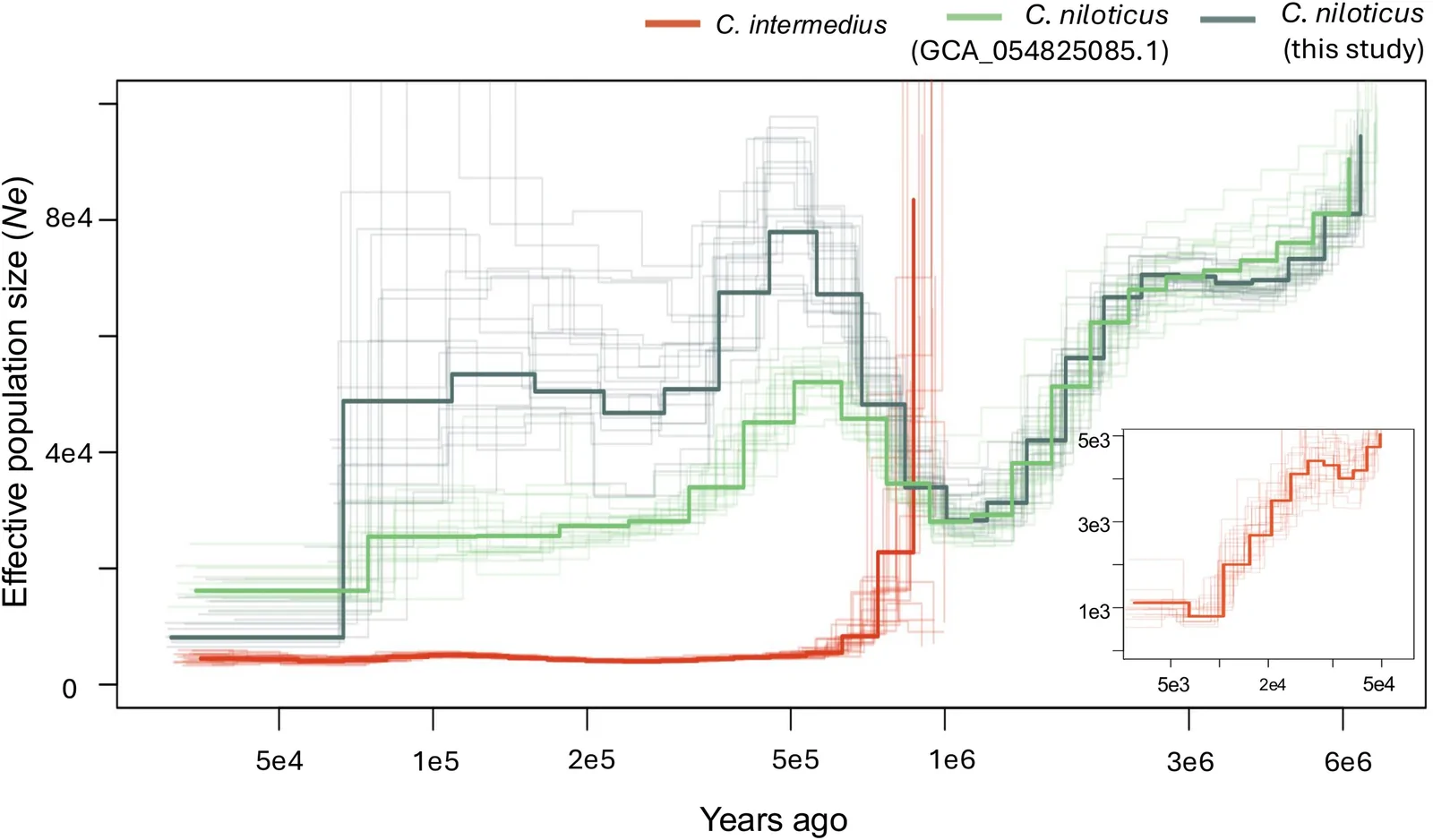
Demographic history inferred from MSMC method for *Crocodylus intermedius* and *C. niloticus*. The inset panel shows a magnified view of the recent demographic trajectory of *C. intermedius*.

The demographic history revealed marked differences in *N*_e_ trajectories and overall, *C. intermedius* showed substantially smaller effective population sizes across most of the inferred time intervals compared to *C. niloticus* (Fig. 4). *N*_e_ for *C. intermedius* ranged from 907 individuals at its minimum (7,304–11,911 yrs ago; ya) to approximately 5,726 individuals during intervals between ∼110,000 and ∼132,000 ya (Supplementary Table 3). For most intervals younger than ∼700,000 ya, *N*_e_ remained relatively stable between ∼4,500 and 5,700 individuals. This stability persisted until roughly 23,000–17,000 ya, when *N*_e_ declined to around 3,000 individuals, followed by a further decrease to 1,265 individuals in the most recent interval (∼3,300 ya). At deeper timescales (>700,000 ya), *N*_e_ appears to increase markedly; however, this pattern likely reflects the limited resolution of coalescent-based methods at very deep timescales when the number of informative SNPs is low (Li and Durbin 2011; Schiffels and Durbin 2014; Hilgers et al. 2025).

In contrast both *C. niloticus* genomes showed substantially larger effective population sizes across comparable intervals. For our genome, *N*_e_ ranged from approximately 9,132 individuals in the most recent interval (0–34,683 ya) to values between ∼52,000 and ∼88,000 individuals (∼243,000–633,000 ya). The GCA_054825085.1 *C. niloticus* genome showed a broadly similar demographic trajectory but with *N*_e_ estimated at approximately 18,216 individuals in the most recent interval (0–38,766 ya). *N*_e_ increased to ∼30,000–38,000 individuals between ∼137,000 and ∼456,000 ya and further rose to ∼50,000–59,000 individuals during the interval spanning ∼456,000–708,000 ya. For both genomes, estimates then gradually declined to approximately 31,000 individuals between ∼1.1 and ∼1.3 million ya, before increasing again at deeper timescales.

### Molecular evolution and selection analyses

Using OrthoFinder-based ortholog inference and codon alignments reconstructed with PAL2NAL (Suyama et al. 2006), we generated a filtered comparative dataset across 6 crocodilian species (*A. sinensis, C. rhombifer, C. intermedius, C. niloticus, C. siamensis,* and *C. porosus*). Ortholog inference identified 11,222 orthologous genes from the initial set of ∼20,380 genes present in the genome annotations. The high level of duplicated BUSCO genes was addressed through isoform filtering, which removed duplicated isoforms from the annotations of all 6 genomes included in the study (Supplementary Table 4). After filtering, we generated a concatenated alignment comprising 5,623 genes, with a total length of 9,952,647 base pairs across the 6 crocodilian species included in the analysis.

### Genome-wide d_N_/d_S_ analyses

**Fig. 5.**
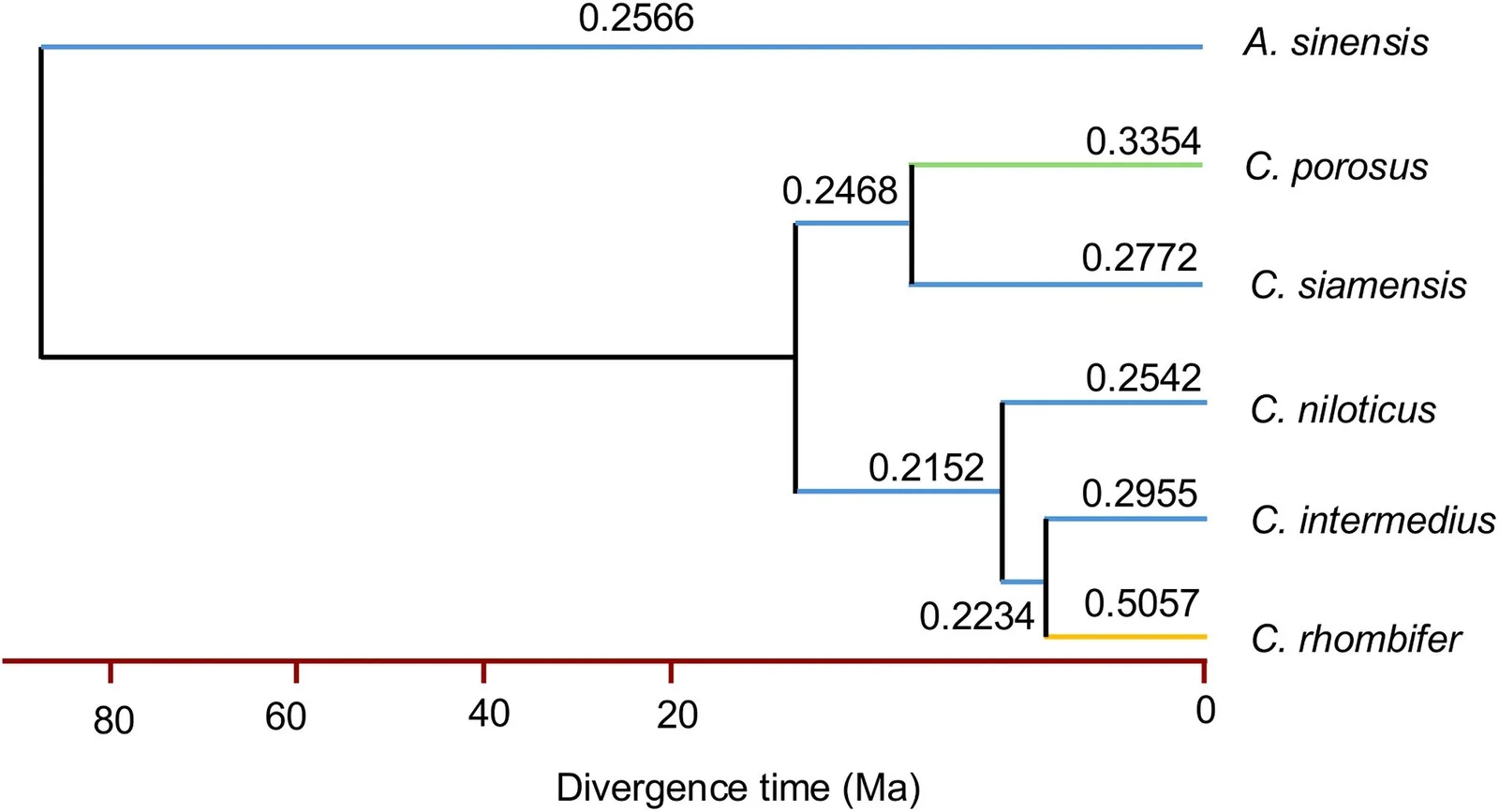
Estimated *d*_N_/*d*_S_ values for the 6 crocodilian species. Branch lengths correspond to divergence time from (Oaks 2011; Shirley et al. 2014; Srikulnath et al. 2015). Numbers indicate the *d*_N_/*d*_S_ values estimated for each lineage under the Free Ratio model for each species, and branch colors represent branches that fall within the same range of *d*_N_/*d*_S_ values.

Using concatenated codon alignments and CODEML likelihood models (Yang 2007), we estimated genome-wide patterns of protein evolution across the 6 crocodilian species. Using the M0 model, which assumes a homogeneous *d*_N_/*d*_S_ ratio across all branches, we estimated an overall ω of 0.26824 (*d*_N_ = 0.0229; *d_S_* = 0.0853; lnL = −14,665,030.417475; np = 12). The FR model, allowing independent *d*_N_/*d*_S_ estimates for each lineage, showed a significantly better fit (lnL = −14,664,141.333649; np = 19; χ^2^ = 1778.17, degrees of freedom = 7, *P* ≪ 0.001), indicating substantial heterogeneity in protein evolution rates among species. The estimated *d*_N_/*d*_S_ values ranged from 0.254236 (*C. niloticus*) to 0.50578 (*C. rhombifer*) (Fig. 5). The results indicated statistically significant differences in *d*_N_/*d*_S_ distributions for all species pairs except between *A. sinensis* and *C. intermedius* (W = 4,120,216; *P* = 0.2416), and between *C. intermedius* and *C. rhombifer* (W = 2,851,169.5; *P* = 0.2850) (Supplementary Table 7).

#### Branch-site tests for positive selection

**Fig. 6.**
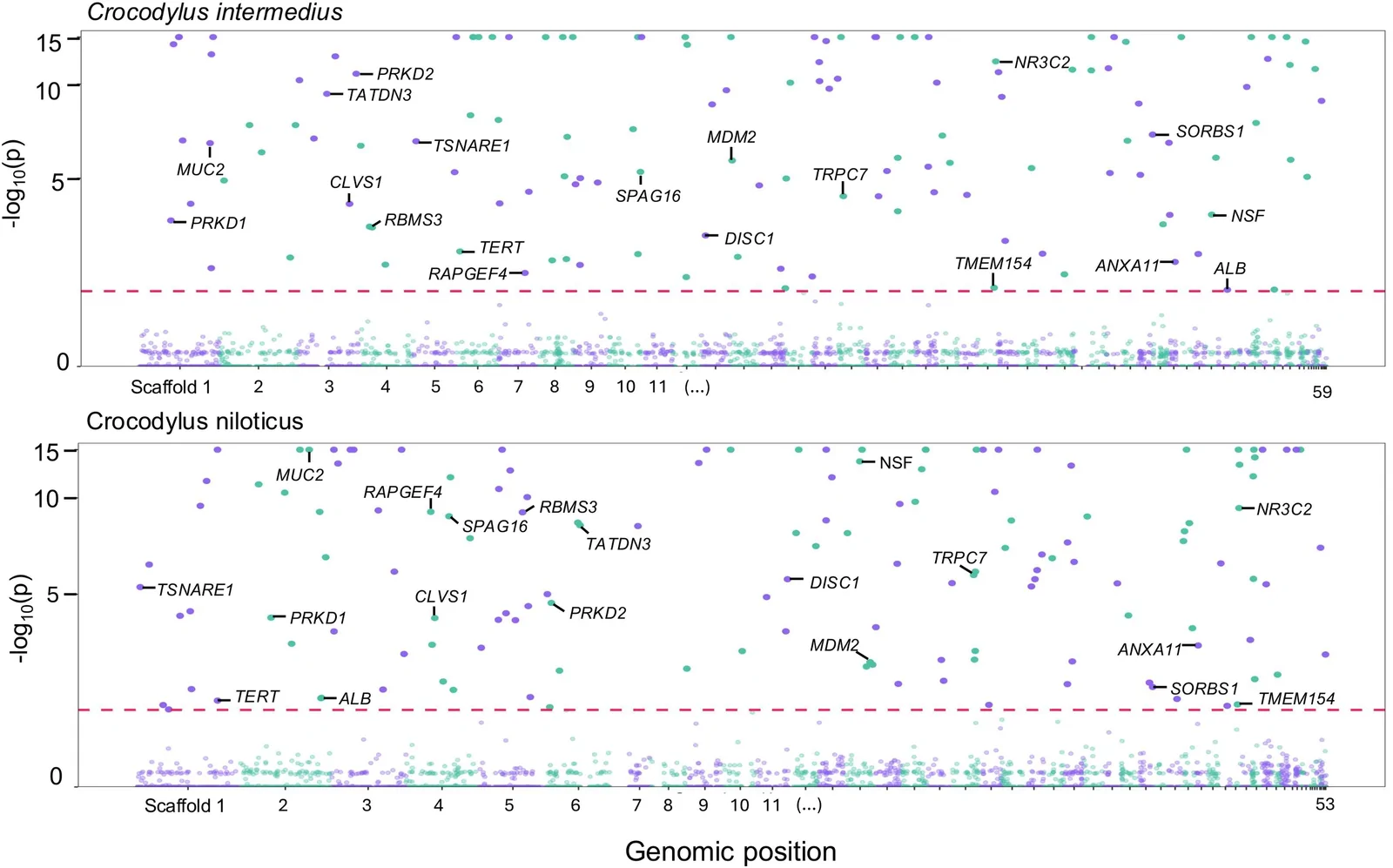
Candidate genes under positive selection across the genomes of *Crocodylus intermedius* and *C. niloticus*. The dashed line indicates the significance threshold (*P* < 0.01) from likelihood ratio tests. Names represent gene ontology terms.

Using CODEML branch-site models (Yang 2007) applied independently to each ortholog alignment, we identified candidate genes evolving under lineage-specific positive selection. Branch-site tests identified 169 candidate genes under positive selection in *C. niloticus* and 158 in *C. intermedius* (*P* < 0.01; Fig. 6; Supplementary Table 8). Among these, 20 genes were shared between species, of which 19 were successfully assigned to orthologous gene symbols based on the *G. gallus* genome (Fig. 6). Overall, both species showed similar functional profiles. Functional annotation using the PANTHER classification system showed that candidate positively selected genes in both species were mainly associated with the biological process categories shown in Supplementary Fig. 4, including cellular process (GO:0009987; 49.0% in *C. intermedius*; 42.2% in *C. niloticus*) and biological regulation (GO:0065007; 28.6%; 23.5%), followed by localization (GO:0051179; 17.7%; 14.7%), response to stimulus (GO:0050896; 12.2%; 13.7%), and metabolic process (GO:0008152; 13.6%; 16.7%). A substantial proportion of genes lacked functional assignment, particularly in *C. niloticus* (40.2% vs 34.0% in *C. intermedius*). At the molecular function level (Supplementary Fig. 4), the most represented categories were binding (GO:0005488; 33.3%; 22.5%), catalytic activity (GO:0003824; 19.7%; 14.7%), and transporter activity (GO:0005215; 9.5%; 5.9%), with a higher proportion of unclassified genes in *C. niloticus* (55.9% vs 36.1% in *C. intermedius*).

## Discussion

### Genome assembly and annotation

The de novo assemblies presented here represent the first published reference-grade genome assemblies for true crocodiles (genus *Crocodylus*). Previously published assemblies for *Crocodylus porosus* (Ghosh et al. 2020) and *C. rhombifer* (Meredith et al. 2024) were generated using short-read data. In addition, we generated the first PCR-free mitochondrial genome assemblies for *C. intermedius* and *C. niloticus*, providing reference-quality genomic resources for comparative and conservation genomics. Although the *C. intermedius* genome was sequenced at higher coverage than *C. niloticus* (48.87× vs 32.63×), its final assembly remained more fragmented (781 contigs vs 556 in *C. niloticus*), likely reflecting differences in input DNA quality. Nevertheless, both assemblies achieved similarly high completeness, with BUSCO scores ranging from 97.0 to 97.9% in *C. intermedius* and from 97.0 to 97.8% in *C. niloticus.* Genome annotation relied on transcriptome-derived evidence from *A. sinensis* and *C. porosus*, which may limit the identification of lineage-specific genes and likely contributed to the BUSCO completeness values recovered for the predicted proteomes (87.2% for *C. intermedius* and 86.8% for *C. niloticus*).

### Synteny and genome architecture

Across pairwise comparisons, we recovered hundreds of large syntenic blocks with median sizes exceeding 3 Mb, indicating strong conservation of genome collinearity between *Alligator* and *Crocodylus* despite ∼80–100 million yrs of divergence (Oaks 2011). Although chromosome-level scaffolding is not yet available for *C. intermedius*, the strong conservation observed between *A. sinensis* and *C. niloticus* suggests that a similar macro-syntenic structure is likely retained in this species. We identified several candidate chromosomal rearrangements, particularly affecting *C. niloticus* chromosomes 1 and 6 and *A. sinensis* chromosome 7, suggesting localized hotspots of structural reorganization. However, the relatively limited number of inferred rearrangements is consistent with previous studies reporting unusually slow rates of structural genome evolution across crocodilians relative to other vertebrates (Green et al. 2014; Chong et al. 2014).

### Demographic history inference

The demographic trajectories inferred here are broadly consistent with previous PSMC-based reconstructions in crocodilians, which similarly report relatively small long-term effective population sizes (Green et al. 2014; Pan et al. 2025). The demographic pattern observed in *C. intermedius* closely mirrors that reported for *A. sinensis* by Pan et al. (2025), as both species exhibit persistently low *N*_e_ across most of their reconstructed history and reduced resolution at deeper timescales (>∼1 million ya). Both species are characterized by historically restricted geographic distributions and are currently classified as Critically Endangered by the International Union for the Conservation of Nature (IUCN 2023a).

The demographic reconstruction inferred for *C. intermedius* indicates persistently low effective population sizes throughout most of the late Quaternary, consistent with prolonged exposure to strong genetic drift in a geographically restricted lineage (Frankham 1996). The pronounced demographic contraction between ∼30,000 and ∼7,300 ya broadly coincides with the transition from the Late Pleistocene to the early Holocene, a period characterized by major climatic and hydrological reorganization across northern South America (Marchant et al. 2001). Paleoecological records from the Colombian Llanos indicate expansion of open grasslands and reduced representation of gallery forests between ∼11.9 and 7.3 ka, followed by wetter mid-Holocene conditions and progressive forest expansion after ∼7,000 ya (van der Hammen and González 1960; Wijmstra and van der Hammen 1966; Berrío et al. 2002; Mayle and Power 2008). These environmental changes likely reduced connectivity among riparian habitats and wetlands, potentially contributing to the demographic contraction inferred here given the strong dependence of *C. intermedius* on riverine systems.

Overall, the demographic trajectory reconstructed for *C. intermedius* suggests that the species has maintained relatively small effective population sizes over extended evolutionary timescales, likely increasing vulnerability to genetic erosion well before the severe anthropogenic population declines documented during the twentieth century (Medem 1981). These long-term patterns are consistent with recent microsatellite-based estimates reporting extremely low contemporary *N*_e_ in the same wild population from which the sequenced individual originated (Castillo-Rodríguez et al. 2024), reinforcing evidence of ongoing genetic vulnerability in the species. Together, the combination of historically low *N*_e_, restricted geographic distribution, and recent population declines highlights the importance of conservation strategies aimed at maintaining the remaining genetic diversity of *C. intermedius* populations.

In contrast, *C. niloticus* exhibited substantially larger inferred coalescent *N*_e_ values, consistent with its broad geographic distribution and ecological flexibility across African freshwater systems. Connectivity among major river basins, including the Nile, Niger, Congo, and Zambezi systems, may have facilitated persistence through periods of climatic fluctuation by allowing populations to track suitable aquatic habitats through time. Despite differences in the magnitude of inferred *N*_e_ between the 2 *C. niloticus* genomes, both individuals showed concordant broad-scale demographic trajectories, including evidence of population expansion between ∼250,000 and ∼700,000 ya. This interval broadly overlaps with periods of increased hydrological connectivity across African freshwater systems associated with intensified monsoon dynamics and expanded lacustrine habitats (Trauth et al. 2009; Tierney et al. 2017).

The demographic shifts inferred around ∼75,000 ya broadly coincide with climatic transitions associated with Marine Isotope Stage (MIS) 4–5 boundary conditions and increasing aridity across Africa (Larrasoaña et al. 2013). However, because MSMC estimates reflect temporal changes in coalescent rates rather than direct reconstructions of census population size, inferred fluctuations may also reflect changes in migration dynamics and population structure (Bansal and Nichols 2025). Differences between individuals may additionally reflect regional demographic histories or technical factors related to sequencing depth, assembly quality, masking strategies, and genome-wide heterozygosity (Hilgers et al. 2025). Nevertheless, the broad concordance between both genomes supports the robustness of the long-term demographic patterns inferred here.

### Molecular evolution and selection analyses

#### Genome-wide d_N_/d_S_ analyses

The genome-wide ω estimates fall within the range previously reported for crocodilians and other long-lived vertebrates (∼0.20–0.35; Green et al. 2014; Figuet et al. 2016; Luzuriaga-Neira and Alvarez-Ponce 2022), consistent with strong long-term purifying selection across the clade, while also reflecting lineage-specific differences in the efficiency of purifying selection among closely related crocodilian lineages. Comparative studies indicate that genome-wide *d*_N_*/d*_S_ ratios are strongly influenced by long-term effective population size and the efficiency of purifying selection rather than mutation rate differences alone, with longevity and age at sexual maturity emerging as important predictors of substitution rate variation (Figuet et al. 2016; Luzuriaga-Neira and Alvarez-Ponce 2022). Consistent with this expectation, *C. intermedius* exhibited slightly higher ω values than *C. niloticus*, paralleling the demographic differences inferred from MSMC analyses. Among the species evaluated, the lowest ω values were observed in the African lineage *C. niloticus*, whereas higher values characterized American and Indo-Pacific representatives of *Crocodylus*. This pattern is broadly consistent with hypotheses proposing an African origin and diversification center for the genus (Shirley et al. 2014; Hekkala et al. 2021), under which dispersal-derived lineages may have experienced stronger long-term demographic reductions associated with founder events. However, this interpretation should be further evaluated using phylogenomic divergence-time frameworks and broader taxonomic sampling.

Under nearly neutral expectations, persistently small effective population sizes reduce the efficacy of purifying selection, facilitating the fixation of slightly deleterious nonsynonymous substitutions and ultimately contributing to elevated genome-wide *d*_N_*/d*_S_ ratios (Ohta 1973). Over evolutionary timescales, these conditions have also been associated with increased intrinsic extinction risk and reduced adaptive potential (Figuet et al. 2016; Luzuriaga-Neira and Alvarez-Ponce 2022). Particularly high ω values observed in *C. rhombifer* are therefore consistent with expectations for lineages experiencing long-term demographic reductions and restricted geographic distributions.

#### Branch-site tests for positive selection

Candidate positively selected genes in both species were primarily associated with ion transport, endocrine regulation, vesicle trafficking, and cellular signaling pathways. These functional categories broadly match patterns previously reported in *C. porosus*, particularly pathways related to membrane transport, osmoregulatory balance, metabolic regulation, and stress response (Ghosh et al. 2020), but differ from those described for *A. sinensis*, where positively selected genes were mainly associated with diving physiology, immune response, and freshwater ecological adaptation (Wan et al. 2013). The presence of *NR3C2*, a key regulator of sodium and water balance, and *TRPC7*, a calcium-permeable ion channel, further supports the hypothesis that ionic and osmotic regulation represents a shared target of adaptive evolution across multiple lineages of the genus. Additionally, the detection of *TERT* among shared candidate genes raises the possibility that selection may also act on pathways related to cellular maintenance and longevity and are closely associated with telomere maintenance mechanisms across vertebrates (Olsson et al. 2018).

The large fraction of unclassified genes highlights current limitations in functional annotation for non-model reptiles and suggests that part of the adaptive signal may involve poorly characterized or lineage-specific genes. Accordingly, the candidate genes identified here should be interpreted as a preliminary framework for future comparative and functional analyses rather than as a complete inventory of adaptive evolution in *Crocodylus.* Future analyses incorporating broader taxonomic sampling, enrichment tests, and gene-level ecological interpretation will be essential to determine whether the lineage-specific candidates identified in *C. intermedius* and *C. niloticus* reflect adaptation to contrasting habitats.

## Conclusion

Overall, this study provides reference-grade long-read genomes for true crocodiles, establishing a comparative framework for genome evolution, demographic history, and molecular adaptation across Crocodylia. Despite strong structural conservation between genomes, we found substantial differences in long-term effective population size and lineage-specific molecular evolution, with consistently lower effective population sizes in *C. intermedius* pointing to a link between demographic history and genome-wide evolutionary dynamics. These resources provide a foundation for broader questions in vertebrate evolutionary biology, including how demographic decline shapes molecular evolution, and will support future work on structural variation, adaptation, and comparative and population genomics in reptiles and other non-model vertebrates with a particular value for conservation genomics in critically endangered species such as *C. intermedius.*

## Supplementary Material

jkag178_Supplementary_Data

## Data Availability

The de novo nuclear genome assemblies and annotated mitochondrial genomes generated in this study are publicly available in the NCBI databases under BioProject accession PRJNA1449441. The nuclear genome assemblies correspond to BioSample accessions SAMN57121639 (*Crocodylus intermedius*) and SAMN57121648 (*Crocodylus niloticus*), with associated sequencing reads available under SRA accession SRR37958024 (*C. intermedius*) and SRR37958025 (*C. niloticus*). The mitochondrial genome assemblies are available in GenBank under accession numbers PZ253398 (*C. intermedius*) and PZ253399 (*C. niloticus*). A complete list of software tools and versions used throughout the analyses is provided in Supplementary Table 9. Scripts used for genome assembly and annotation are available at GitHub https://github.com/Agustol/GenomeAssemblyAndAnnotation, and scripts associated with molecular evolution analyses are available at GitHub https://github.com/Agustol/PAML_analyses. Supplemental material available at G3 online.
