# Supplementary material for: Genome evolution and long-term demographic history in true crocodiles
Source: G3 (Bethesda). 2026 Aug 5;16(9):jkag178. doi: 10.1093/g3journal/jkag178 (PMC13535408; doi:10.1093/g3journal/jkag178)
Supplement: Supplementary file 1 [file jkag178_supplementary_data.zip › Supplemental_Material_G3-2026-407008.pdf]

SUPPLEMENTARY FILES

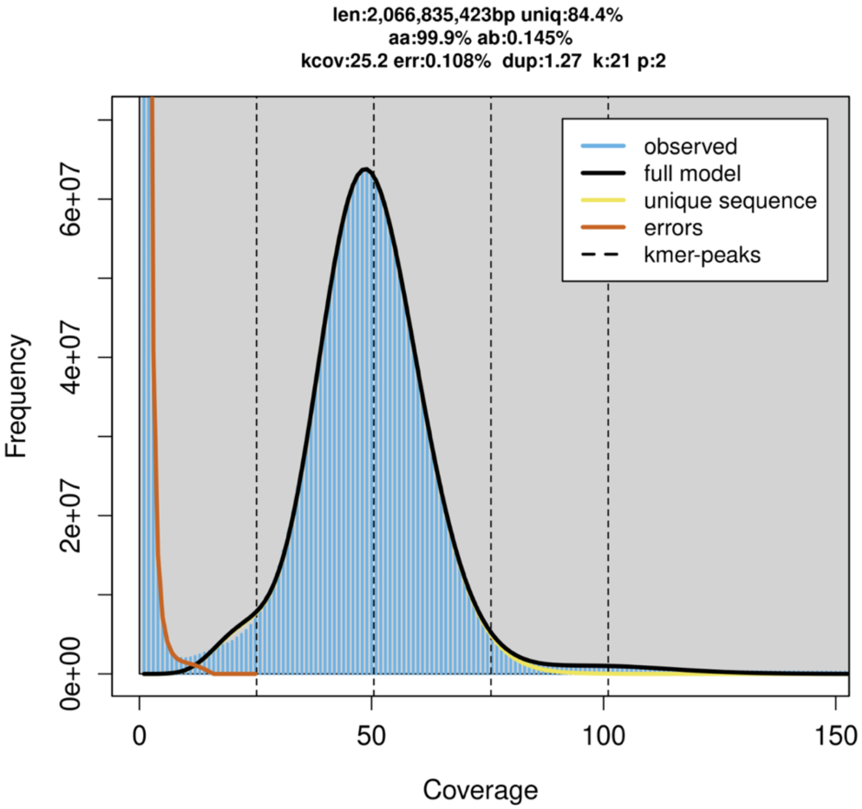

Figure S1. GenomeScope profile inferred from *k*-mer frequency analysis of PacBio HiFi reads for *Crocodylus intermedius.* The histogram shows the observed *k*-mer coverage distribution (blue), with the fitted GenomeScope model overlaid (black). The analysis estimated a haploid genome size of ~2.07 Gb, with 84.4% unique sequence content and a low heterozygosity rate (ab = 0.145%, representing the proportion of heterozygous sequence), consistent with a highly homozygous genome (aa = 99.9%, representing the proportion of homozygous sequence). The main *k*-mer peak at ~25× coverage corresponds to the expected haploid coverage, consistent with an overall sequencing depth of approximately 50× for this diploid genome. Low-coverage *k*-mers represent sequencing errors (orange). Dashed vertical lines indicate inferred *k*-mer peaks used by the model. The estimated sequencing error rate was 0.108%, and the duplication rate was low (1.27%).

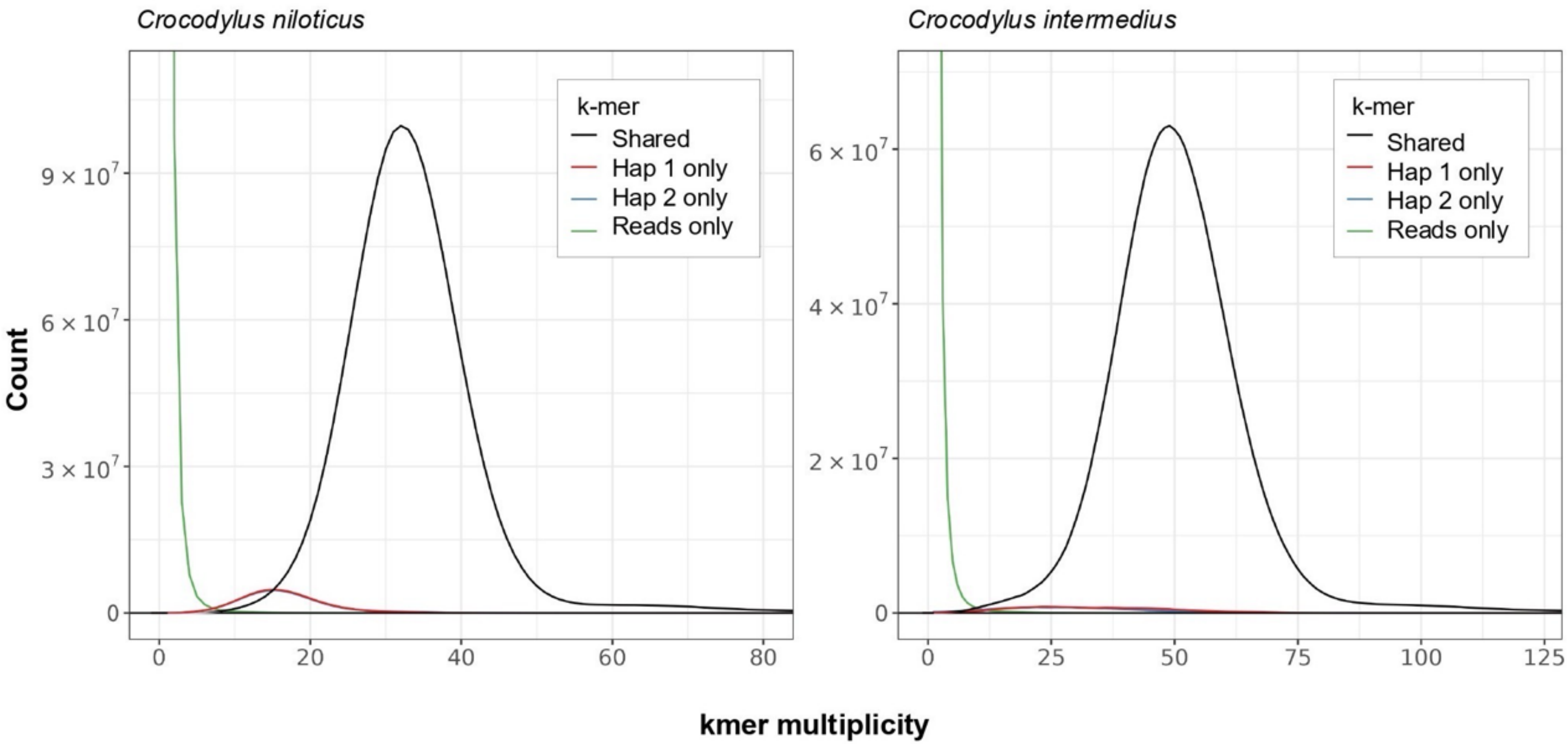

Figure S2. Merqury *k*-mer spectra plots showing assembly completeness and haplotype representation.

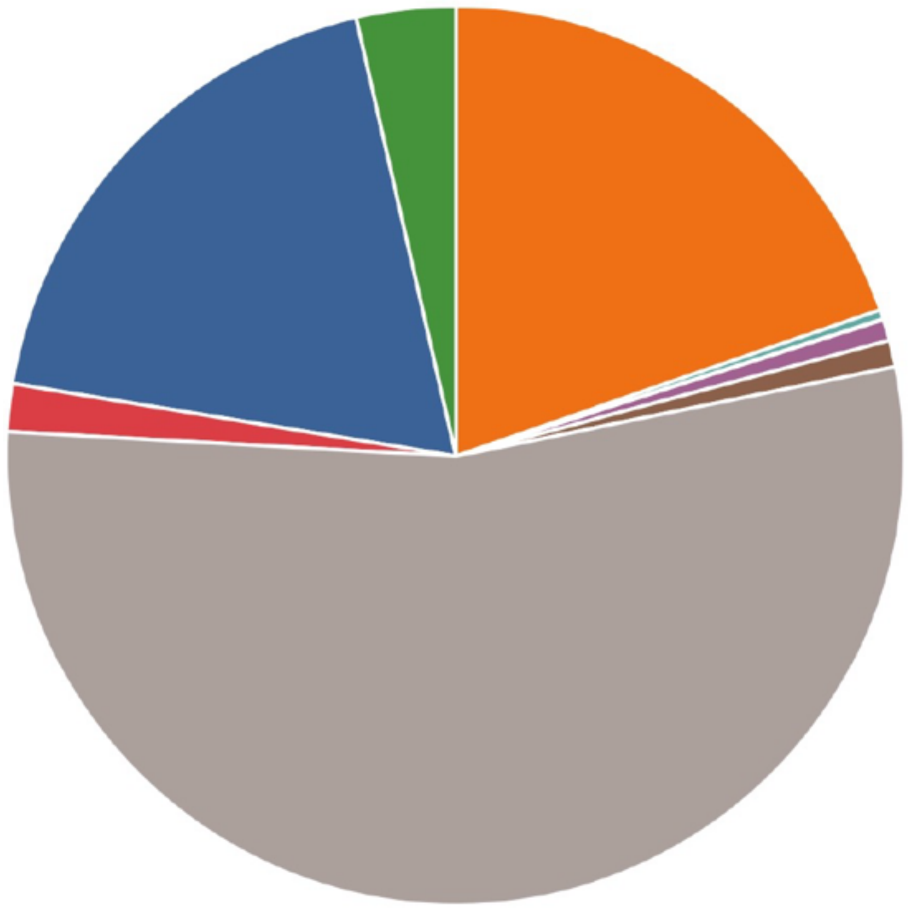

TE class	% in *C. intermedius*	% in *C. niloticus*	% difference
LINE	18.85	17.52	−1.33
Unclassified	19.73	18.56	−1.17
DNA	3.57	3.58	≈0.00
LTR	1.71	1.84	+0.13
SINE	0.34	0.32	−0.02
Penelope	0.77	0.86	+0.09
Rolling Circle	0.02	0.04	+0.01
Other (Simple Repeat, RNA, etc.)	0.95	2.08	+1.13
Non-Repeat	54.05	55.20	+1.15

Figure S3. Genome-wide comparison of transposable element classes in *Crocodylus intermedius* and *C. niloticus*.

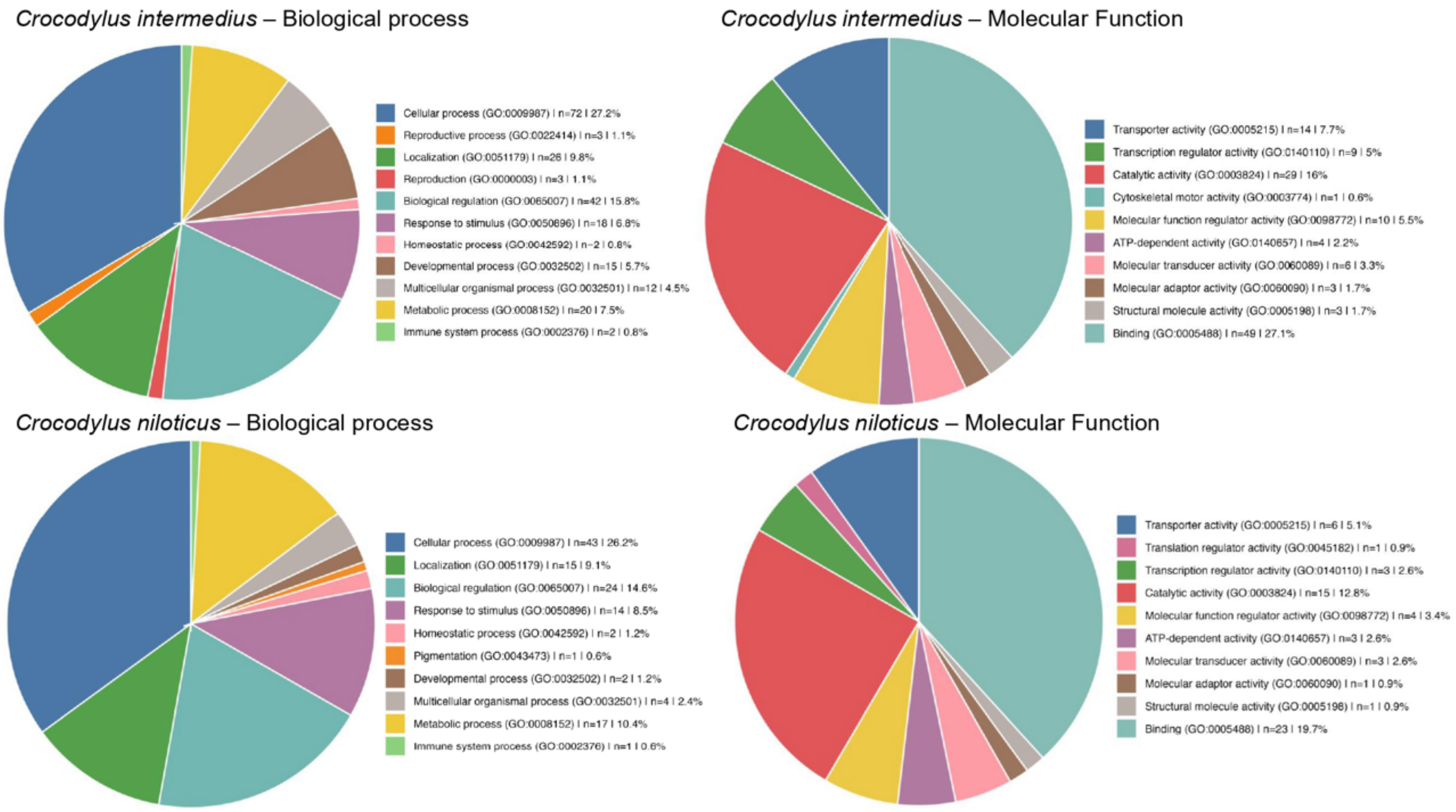

Figure S4. Functional classification of candidate genes under positive selection in *Crocodylus intermedius* and *C. niloticus* based on PANTHER Gene Ontology categories.

Table S1. QUAST summary statistics for the genome assemblies of *Crocodylus intermedius* and *C. niloticus*.

	Crocodylus intermedius **primary assembly**	***Crocodylus niloticus*** **primary assembly**
**# contigs (>= 0 bp)**	781	556
**# contigs (>= 10000 bp)**	781	550
**# contigs (>= 25000 bp)**	766	488
**# contigs (>= 50000 bp)**	568	360
**Total length (>= 0 bp)**	2,353,064,391	2,352,358,014
**Total length (>= 10000 bp)**	2,353,064,391	2,352,305,746
**Total length (>= 25000 bp)**	2,352,724,666	2,351,126,487
**Total length (>= 50000 bp)**	2,345,102,755	2,346,621,333
**# contigs**	781	556
**Largest contig**	138,874,091	174,938,404
**Total length**	2,353,064,391	2,352,358,014
**GC (%)**	45	45
**N50**	48,683,327	61,736,884
**N90**	16,813,806	19,478,582
**auN**	60,720,575	80,409,406.0
**L50**	15	11
**L90**	44	37
**# N's per 100 kbp**	0	0

Table S2. BUSCO assessment (using sauropsida_odb10 database) of newly annotated *Crocodylus intermedius* and *C. niloticus* genomes based on combined *Alligator sinensis* and *C. porosus* transcriptomic evidence (longest isoform retained), and of published *A. sinensis* (Pan et al. 2025) and *C. porosus* (Ghosh et al. 2020) proteomes (Total BUSCOs = 7480)

**Species**	**Data type / annotation**	**Complete BUSCOs**	**Single-copy**	**Duplicated**	**Fragmented**	**Missing**
C. intermedius	PacBio, longest isoform	6595 (88.2%)	6521 (87.2%)	74 (1.0%)	145 (1.9%)	740 (9.9%)
C. niloticus	PacBio, longest isoform	6569 (87.8%)	6493 (86.8%)	76 (1.0%)	137 (1.8%)	774 (10.4%)
C. porosus	Short reads, published proteome	7250 (96.9%)	4325 (57.8%)	2925 (39.1%)	37 (0.5%)	193 (2.6%)
A. sinensis	Long reads, published proteome	7317 (97.9%)	4014 (53.7%)	3303 (44.2%)	85 (1.1%)	78 (1.0%)

TS3. MSMC-inferred effective population size (N_e) trajectories for *Crocodylus intermedius* and *C. niloticus*. Start and end times correspond to the boundaries of each MSMC time interval. "Ne all runs" represents estimates obtained using the full genome, whereas "Ne min" and "Ne max" correspond to the minimum and maximum values across 20 independent MSMC runs using randomly selected 30% genome subsets.

	***C. intermedius* (this study)**					***C. niloticus* (this study)**					***C. niloticus* (Genbank GCA_054825085.1)**				
**Interval**	**Start years**	**End years**	**Ne all runs**	**Ne min**	**Ne max**	**Start years**	**End years**	**Ne all runs**	**Ne min**	**Ne max**	**Start years**	**End years**	**Ne all runs**	**Ne min**	**Ne max**
**0**	0.00	3,366.40	1,265.07	718.41	2,029.35	0.00	34,683.43	9,132.38	6,643.87	18,750.96	0.00	38,766.57	18,216.14	14,613.82	25,278.09
**1**	3,366.40	7,304.26	1,265.07	718.41	2,029.35	34,683.43	75,254.57	9,132.38	6,643.87	18,750.96	38,766.57	84,114.00	18,216.14	14,613.82	25,278.09
**2**	7,304.26	11,910.57	907.55	654.52	1,707.30	75,254.57	122,712.29	55,010.22	30,754.65	187,816.76	84,114.00	137,158.86	28,722.62	24,039.64	45,646.71
**3**	11,910.57	17,298.80	2,278.14	1,158.53	3,299.59	122,712.29	178,226.29	60,213.76	41,245.91	94,067.87	137,158.86	199,208.29	28,848.26	22,624.59	37,893.78
**4**	17,298.80	23,601.69	3,052.42	2,281.25	4,491.56	178,226.29	243,163.71	56,900.23	51,542.35	86,373.85	199,208.29	271,790.57	30,767.49	25,486.37	37,556.74
**5**	23,601.69	30,974.57	3,970.46	2,948.22	6,086.12	243,163.71	319,122.86	52,718.31	36,597.79	80,160.27	271,790.57	356,694.29	31,715.73	29,197.25	45,822.14
**6**	30,974.57	39,598.86	4,676.76	3,336.12	6,641.15	319,122.86	407,980.00	57,321.24	38,894.68	89,461.16	356,694.29	456,008.57	38,293.96	31,381.26	57,651.65
**7**	39,598.86	49,687.14	5,022.08	3,480.44	6,203.70	407,980.00	511,917.14	76,077.44	51,850.67	106,019.59	456,008.57	572,182.86	50,881.94	39,472.94	63,504.78
**8**	49,687.14	61,488.00	4,905.51	4,020.55	5,934.97	511,917.14	633,497.14	87,825.72	67,236.05	109,198.69	572,182.86	708,077.14	58,723.22	51,025.10	62,569.06
**9**	61,488.00	75,292.00	4,555.25	4,125.02	5,505.44	633,497.14	775,717.14	75,751.88	55,794.94	90,031.53	708,077.14	867,040.00	51,596.45	44,001.02	62,265.80
**10**	75,292.00	91,439.14	4,770.43	3,843.20	5,976.10	775,717.14	942,080.00	54,360.47	40,989.14	64,946.06	867,040.00	1,052,988.57	38,994.50	34,348.27	52,716.43
**11**	91,439.14	110,327.43	5,389.29	4,126.76	6,332.78	942,080.00	1,136,680.00	38,291.08	34,148.22	47,870.68	1,052,988.57	1,270,500.00	31,645.61	28,846.15	57,574.63
**12**	110,327.43	132,421.71	5,725.74	4,819.66	6,166.53	1,136,680.00	1,364,314.29	31,859.59	29,372.94	44,663.24	1,270,500.00	1,524,934.29	32,936.28	27,126.00	70,594.31
**13**	132,421.71	158,266.86	5,540.28	5,142.05	6,055.76	1,364,314.29	1,630,591.43	35,284.00	28,368.30	57,499.09	1,524,934.29	1,822,557.14	42,955.25	27,408.67	80,934.67
**14**	158,266.86	188,498.86	5,147.78	4,718.80	6,038.13	1,630,591.43	1,942,068.57	47,393.77	33,322.05	72,225.65	1,822,557.14	2,170,702.86	57,834.56	35,528.73	85,989.76
**15**	188,498.86	223,863.14	4,825.28	4,429.77	5,689.83	1,942,068.57	2,306,417.14	63,372.08	45,939.17	81,390.35	2,170,702.86	2,577,945.71	70,265.67	49,109.05	88,572.80
**16**	223,863.14	265,230.29	4,589.08	4,387.88	5,151.59	2,306,417.14	2,732,614.29	75,181.08	61,873.27	84,436.71	2,577,945.71	3,054,314.29	76,655.64	61,026.76	91,394.06
**17**	265,230.29	313,620.00	4,495.70	4,324.94	5,011.12	2,732,614.29	3,231,171.43	79,520.32	73,580.47	84,875.43	3,054,314.29	3,611,542.86	79,059.21	67,711.41	94,965.65
**18**	313,620.00	370,222.86	4,653.87	4,410.86	5,284.36	3,231,171.43	3,814,342.86	79,161.90	76,428.75	83,635.14	3,611,542.86	4,263,371.43	80,372.05	71,165.14	99,578.91
**19**	370,222.86	436,434.29	4,971.02	4,305.98	5,593.58	3,814,342.86	4,496,485.71	77,920.69	75,042.07	85,815.20	4,263,371.43	5,025,857.14	82,312.70	71,653.44	105,537.28

**20**	436,434.29	513,885.71	5,261.42	4,364.86	6,310.54	4,496,485.71	5,294,457.14	78,508.12	74,137.64	91,552.37	5,025,857.14	5,917,771.43	85,739.01	73,318.43	114,835.50
**21**	513,885.71	604,482.86	5,544.58	4,734.85	8,147.86	5,294,457.14	6,227,885.71	82,607.23	75,735.92	99,317.08	5,917,771.43	6,961,085.71	91,373.48	77,992.62	133,247.79
**22**	604,482.86	710,460.00	6,182.47	5,071.67	13,655.26	6,227,885.71	7,319,742.86	91,339.13	82,616.33	111,110.66	6,961,085.71	8,181,485.71	101,949.21	86,910.19	173,445.18
**23**	710,460.00	834,428.57	9,363.40	5,390.23	36,885.87	7,319,742.86	8,596,942.86	106,379.74	96,595.71	134,561.61	8,181,485.71	9,609,057.14	127,892.45	103,945.56	268,749.25
**24**	834,428.57	979,440.00	25,674.06	6,133.33	150,378.59	8,596,942.86	10,090,971.43	131,892.40	118,437.92	190,037.07	9,609,057.14	11,278,942.86	197,230.41	139,638.40	485,601.54
**25**	979,440.00	1,149,065.71	94,037.80	8,462.02	594,617.96	10,090,971.43	11,838,600.00	172,247.78	145,278.02	283,787.76	11,278,942.86	13,232,314.29	351,908.22	199,942.97	847,882.68
**26**	1,149,065.71	1,347,485.71	200,675.31	13,932.14	929,888.37	11,838,600.00	13,882,885.71	212,622.37	168,191.37	361,910.55	13,232,314.29	15,517,285.71	513,081.00	265,165.11	1,026,316.83
**27**	1,347,485.71	1,579,588.57	17,046.33	7,784.56	24,765.81	13,882,885.71	16,274,171.43	101,424.45	68,919.21	202,060.16	15,517,285.71	18,190,114.29	123,877.82	90,091.25	203,784.62
**28**	1,579,588.57	1,851,088.57	17,046.33	7,784.56	24,765.81	16,274,171.43	19,071,400.00	101,424.45	68,919.21	202,060.16	18,190,114.29	21,316,628.57	123,877.82	90,091.25	203,784.62
**29**	1,851,088.57	2,168,677.14	667,787.66	343,629.18	2,697,870.56	19,071,400.00	22,343,457.14	1,018,272.70	529,610.43	2,220,733.72	21,316,628.57	24,973,914.29	2,208,696.17	1,550,078.02	3,271,076.06
**30**	2,168,677.14	2,540,177.14	667,787.66	343,629.18	2,697,870.56	22,343,457.14	26,170,942.86	1,018,272.70	529,610.43	2,220,733.72	24,973,914.29	29,252,000.00	2,208,696.17	1,550,078.02	3,271,076.06
**31**	2,540,177.14	Inf	667,787.66	343,629.18	2,697,870.56	26,170,942.86	Inf	1,018,272.70	529,610.43	2,220,733.72	29,252,000.00	Inf	2,208,696.17	1,550,078.02	3,271,076.06

Table S4. BUSCO completeness statistics for genome annotations after duplicate isoform removal using protein mode and the sauropsida_odb10 dataset (n = 7,480).

**Species**	**Data type**	**Complete BUSCOs**	**Single-copy**	**Duplicated**	**Fragmented**	**Missing**
C. porosus	Duplication removal from original annotation	6630 (88.6%)	6585 (88.0%)	45 (0.6%)	151 (2.0%)	699 (9.4%)
C. siamensis	GeMoMa annotation	6542 (87.5%)	6484 (86.7%)	58 (0.8%)	141 (1.9%)	797 (10.6%)
C. rhombifer	GeMoMa annotation	6617 (88.5%)	6490 (86.8%)	127 (1.7%)	198 (2.6%)	665 (8.9%)
Alligator sinensis	Duplication removal from original annotation	—	—	—	—	—

Table S5. Chromosome-scale candidate structural rearrangement breakpoints between *Crocodylus niloticus* and *Alligator sinensis*. Cn: *Crocodylus niloticus* As: *Alligator sinensis* chr: chromosome

Species	Chromosome	Orientation change	Chromosome switch	Chromosome switch with orientation change	Total breakpoints
Crocodylus niloticus	Cn_chr_1	2	6	0	8
	Cn_chr_2	0	6	0	6
	Cn_chr_4	0	4	0	4
	Cn_chr_5	0	2	0	2
	Cn_chr_6	2	12	2	16
	Cn_chr_7	0	2	0	2
	Cn_chr_10	0	4	0	4
	Cn_chr_12	0	2	0	2
	Cn_chr_15	0	3	0	3
	Cn_chr_16	0	1	0	1
Alligator sinensis	As_chr_1	6	0	0	6
	As_chr_2	5	0	0	5
	As_chr_3	6	1	0	7
	As_chr_4	2	0	0	2
	As_chr_7	9	0	0	9
	As_chr_10	4	0	0	4
	As_chr_11	2	4	1	7
	As_chr_14	3	0	0	3
	As_chr_15	4	0	0	4
	As_chr_16	2	0	0	2

Table S6. Genomic coordinates and orientation of candidate structural breakpoints between *Crocodylus niloticus* and *Alligator sinensis*. Cn: *C. niloticus* As: *A. sinensis* chr: chromosome. Upstream and downstream blocks represent adjacent syntenic regions flanking each breakpoint in the focal genome. The orthologous chromosome columns indicate the corresponding chromosomes in the other genome before and after the breakpoint, allowing identification of inversion and interchromosomal rearrangement events.

See Excel Table

Table S7. Pairwise Wilcoxon signed-rank test results for differences in gene-wise branch-specific d_N/d_S distributions among species. N indicates the number of genes included per species comparison, and W indicates the Wilcoxon test statistic. Significant values shown in bold.

**Species 1**	**N species 1**	**Species 2**	**N species 2**	**W**	**p-value**
C. intermedius	2482	*A. sinensis*	3265	4120216	0.24166669
C. intermedius	2482	*C. niloticus*	2833	3318550.5	**6.80E-05**
C. intermedius	2482	*C. porosus*	3894	5381045.5	**3.22E-16**
C. intermedius	2482	*C. rhombifer*	2482	2851169.5	0.28509858
C. intermedius	2482	*C. siamensis*	3747	5029351	**4.93E-09**
C. niloticus	2833	*A. sinensis*	3265	4261211	**4.99E-09**
C. niloticus	2833	*C. porosus*	3894	4566662	**6.64E-39**
C. niloticus	2833	*C. rhombifer*	2482	3082683	**0.00859422**
C. niloticus	2833	*C. siamensis*	3747	4563781.5	**2.09E-26**
C. porosus	3894	*A. sinensis*	3265	7055632.5	**2.91E-17**
C. porosus	3894	*C. rhombifer*	2482	4958449.5	**1.40E-18**
C. porosus	3894	*C. siamensis*	3747	7056937.5	**0.00951011**
C. rhombifer	2482	*A. sinensis*	3265	3834482.5	**0.00859422**
C. rhombifer	2482	*C. siamensis*	3747	4641399.5	**2.35E-11**
C. siamensis	3747	*A. sinensis*	3265	6576511.5	**7.15E-09**

Table S8. Supplementary Table S1. Branch-site test results for *Crocodylus niloticus* and *C. intermedius* candidate genes under positive selection, including likelihood ratio statistics, Gene Ontology categories, and PANTHER protein family classifications.

See Excel Table

Table S9. Software tools and versions used throughout genome assembly, annotation, orthology inference, and molecular evolution analyses.

Step	Software / Pipeline	Version
k-mer counting	Jellyfish; Meryl	2.3.0; v1.3
Genome size and heterozygosity estimation	GenomeScope	v2.0
De novo genome assembly	Hifiasm	v0.25.0
Assembly quality statistics	QUAST	v5.3.0
Assembly and proteome completeness assessment (single-copy orthologs)	BUSCO (vertebrata_odb10; sauropsida_odb10)	v5.5.0
Reference-free assembly accuracy assessment	Merqury	v1.3
Mitochondrial genome assembly	MitoHiFi	v3.2.3
Mitogenome visualization and manual annotation	Geneious Prime	v2019.2.3
Repeat identification and masking	EarlGrey	v6.2.0
Homology-based genome annotation	GeMoMa	v1.7.1
Filtering of duplicated gene models	AGAT	v1.6.1
Annotation completeness assessment	OMAmer; OMArk	v2.1.0; v0.3.0
Reference-guided scaffolding	RagTag	v2.1.0
Orthology inference	OrthoFinder	v3.1.3
Multiple sequence alignment	MAFFT	v7.525
Codon alignment reconstruction	PAL2NAL	v14.1
Alignments concatenation	AMAS	v1.2
Genome-wide dN/dS estimation and branch-site tests for positive selection	PAML (CODEML; M0, FR, Model A, Model A null)	v4.9g
Functional annotation and Gene Ontology classification	PANTHER	—
